# Legume Intake, Body Weight, and Abdominal Adiposity: 10-Year Weight Change and Cross-Sectional Results in 15,185 U.S. Adults

**DOI:** 10.3390/nu15020460

**Published:** 2023-01-16

**Authors:** Larry A. Tucker

**Affiliations:** College of Life Sciences, Brigham Young University, Provo, UT 84602, USA; tucker@byu.edu; Tel.: +1-801-422-4927

**Keywords:** beans, fiber, pulses, carbohydrate, fat, protein, NHANES, obesity, waist

## Abstract

There were three objectives: (1) evaluate the relationship between legume intake and weight change across the previous 10 years, (2) examine the cross-sectional associations between legume consumption, BMI, and abdominal adiposity, and (3) determine if the relationship between legume intake and the outcomes were influenced by multiple covariates, particularly fiber intake. The sample included 15,185 randomly selected adults representative of the U.S. population. Percent change in weight was used as the outcome measure for the 10-year analysis. BMI, and waist circumference, corrected for height, were employed as the outcomes for the cross-sectional analyses. Legume, fiber, and energy intakes were measured using the average of two 24-h dietary recalls. Legume intake was divided into three categories. Five demographic and five lifestyle covariates were controlled statistically. There was an inverse dose-response relationship between legume intake and percent weight change over the previous 10 years after adjusting for 9 of the covariates (F = 6.5, *p* = 0.0028). However, after controlling for fiber with the other covariates, there were no differences across the three legume intake groups (F = 1.9, *p* = 0.1626). The cross-sectional findings showed similar inverse dose-response results until fiber intake was controlled. Then the associations became non-significant. In conclusion, legume intake is a good predictor of percent weight change over the previous 10 years, and it is also a significant predictor of BMI and abdominal adiposity cross-sectionally. These relationships are strongly influenced by fiber consumption. Evidently, legumes have dietary advantages, especially high fiber levels, that seem to be valuable in the battle against weight gain and obesity.

## 1. Introduction

Legumes have numerous nutritional characteristics that have the potential to decrease the risk of disease [1,2,3]. Meta-analyses show that legume intake is inversely associated with incident cardiovascular disease, including coronary heart disease and cardiometabolic risk factors [1,2,4,5,6]. Despite the many disease-prevention and health-enhancing qualities associated with legume intake, consumption levels tend to be modest in the U.S. and appear to be decreasing [7,8]. Based on purchasing data from grocery stores, chain supermarkets, club stores, and other retail outlets, Semba et al. concluded in 2021, “Although legumes are inexpensive, healthy, and a sustainable source of protein, per capita legume intake remains low in the U.S. and below dietary guidelines” [9].

One of the most common chronic diseases in the United States is obesity. It affects about 43% of the adult population, with another 31% classified as overweight [10]. Research indicates that regular legume consumption may help prevent obesity [11]. Legumes, particularly beans, include only small amounts of dietary fat and large amounts of dietary fiber. Moreover, the glycemic index of beans is low. They also contain high levels of plant protein. They are satiating, and they can enhance the gut microbiome. Consequently, beans and other legumes have many qualities that may help in the fight against weight gain and its consequences [11,12].

Although legumes have many nutritional characteristics that should make them a desirable food when the prevention of obesity is the goal, individual weight loss studies focusing on legumes have rarely resulted in favorable outcomes [13,14]. However, when combined using meta-analysis, results suggest that legume consumption may be a good choice for weight loss [2,14]. Clearly, individual investigations and meta-analyses differ in their findings and in their conclusions. Consequently, Williams et al. determined, “There is insufficient evidence to make clear conclusions about the protective effect of legumes on weight” [15].

More than 20 years ago, Papanikolaou et al. studied the relationship between bean consumption and a variety of outcomes using a cross-sectional design [16]. The present study and the investigation by Papanikolaou et al. have some similarities and many differences. Papanikolaou et al. focused on bean consumption, primarily baked beans, not legume intake. The earlier study compared bean consumers to non-consumers. Intake was not quantified, and weight change over time was not addressed. Only one dietary recall assessment was used, yet the Papanikolaou investigation found that bean eaters had lower body weights and lower waist circumferences than non-consumers [16].

According to the literature, few studies have examined the influence of legume intake on 10-year weight change in a large sample of adults representing the U.S. population. Moreover, investigations focusing on the relationship between legume intake and abdominal adiposity are scarce. Finally, few, if any, studies have examined the mediating influence of dietary fiber on the association between legume intake and measures of body mass and abdominal obesity. Hence, the present investigation was conducted to reduce some of the potential issues identified in other studies. In particular, a large representative sample of the U.S. population was utilized (n = 15,185). Moreover, changes in body mass over 10 years were assessed, and cross-sectional relationships were also examined.

Given previous research, there were three key objectives of this study: (1) evaluate the relationship between legume intake and weight change across the previous 10 years, (2) examine the cross-sectional associations between legume consumption, body mass, and abdominal adiposity, and (3) determine the extent to which the relationship between legume intake and the study outcomes were a result of differences in several covariates, particularly dietary fiber intake.

## 2. Materials and Methods

### 2.1. Study Design and Sample

Study participants were chosen randomly to participate in the U.S. National Health and Nutrition Examination Survey (NHANES). Data were collected by NHANES over 8 years, from 2011 to 2018. Participants were assigned specific sample weights by NHANES based on census outcomes. Consequently, results from the present study can be generalized to the non-institutionalized adult population of the U.S.

The Ethics Review Board for the U.S. National Center for Health Statistics (NCHS) approved the data gathering procedures and publishing of the NHANES data online. The files posted online by NHANES contain no confidential information and are free to the public. The protocol codes indicating approval for NHANES data collected from 2011 to 2018 were: Protocol #2011–17 and Protocol #2018-01. Each subject provided written consent to take part in the national survey.

The present study’s sample size varied based on if the focus was 10-year weight change or cross-sectional outcomes. A total of 10,137 subjects were part of the 10-year analyses, whereas 15,185 individuals were included in the cross-sectional analyses. The difference was because the 10-year analyses included subjects 36–75 years of age, whereas the cross-sectional part of the study included subjects 18–75 years old. If the 10-year analyses had included subjects as young as 18, then their initial body mass would have been based on their weight at 8 years of age. Therefore, a minimum subject age limit was set at 36 years for the 10-year weight change variable, so only fully grown individuals (those ≥26 years old) were included.

Subjects who were underweight (BMI < 18.5) were not included in the sample because of the risk of an eating disorder or severe illness (n = 270). Pregnant women (n = 177) and adults who fasted or who reported extremely low 24-h energy intakes (3 standard deviations or more below the mean: <492 kcal) were also excluded from the analyses (n = 361).

### 2.2. Instrumentation and Measurement Methods

The association between legume intake and 10-year percent weight change and cross-sectional BMI and abdominal adiposity were evaluated. Age, sex, race/ethnicity, economic status, year of assessment, energy intake, total physical activity, cigarette smoking, alcohol use, and fiber intake were used as covariates, with a special focus on fiber intake.

Diet. Legume consumption, fiber intake, and energy consumption were each indexed using the average of two 24-h dietary recall evaluations. Both dietary recalls collected comprehensive data about all the foods and beverages consumed during the 24-h prior to the interview (midnight to midnight). The first of the two dietary interviews were conducted in person in a private room in the NHANES mobile examination center. The second interview was conducted by telephone 3–10 days after the in-person assessment. The diet interview was based on the “What We Eat in America” partnership between the U.S. Department of Agriculture (USDA) and the U.S. Department of Health and Human Services. A variety of measuring guides, such as glasses, mugs, spoons, bowls, bottles, cups, thickness sticks, a ruler, etc., were available to help subjects accurately report food quantities. After finishing the initial in-person diet assessment, participants were given sample bowls, glasses, etc., and a food model booklet to help them during the upcoming telephone dietary evaluation.

The personnel conducting the diet recalls were multi-lingual. Interviewers were directed by scripts, and the computer-based program provided a standard interview process. The diet evaluations utilized a multi-pass format called the Automated Multiple Pass Method (AMPM), available online [17]. This dietary assessment utilized food probes to maximize recall of all foods and beverages consumed. The United States Department of Agriculture (USDA) National Food and Nutrient Database was used to measure food and nutrient content [18]. It is evaluated and updated regularly and stands as the gold standard within the United States [19].

Results derived from the NHANES 24-h diet recall protocol have been studied comprehensively, and predictive validity has been confirmed by many investigations. Many studies have shown that dietary findings derived from the recalls are predictive of a host of chronic and acute conditions [20,21,22,23,24,25].

Harvard nutritional epidemiologist, Walter Willett, indicates that a single 24-h dietary recall assessment is likely sufficient if the sample size is large. Additionally, he states that to estimate within-person variability, “it is statistically most efficient to increase the number of individuals in the sample, rather than to increase the number of days beyond 2 days per individual” (page 55) [26]. Taking into account that the current study included a sample of over 15,000 adults, each completing two 24-h dietary recalls, the assessment strategy utilized was suitable to secure quality estimates of dietary consumption.

Legume Intake. Total legume intake was used as the exposure variable. Because legume consumption tends to increase as total energy intake increases, legume intake was indexed as average grams consumed per 1000 kilocalories (kcal) over the two independent days of dietary evaluation. Included were dried beans (nonspecific), canned beans, chickpeas (nonspecific, dried, or canned), beans from fast-food, bean dip, black-eyed peas, kidney beans, white beans, black beans, fava beans, lima beans, navy beans, pink beans, pinto beans, Peruvian beans, soybeans, split peas, wasabi peas, mung beans, baked beans, refried beans, pork and beans, lentil curry, and other legumes.

Legume intake was divided into 3 categories: None, Low, and Moderate/High. The “None” group included adults reporting no legume consumption during the two dietary recall assessments. The Low group comprised adults reporting some legume consumption, but less than 47 g per 1000 kcal, on average, over the two days of dietary evaluation. The Moderate/High category included men and women who ate 47 g or more of legumes per 1000 kcal, on average, during the two days of dietary assessment. The Low and Moderate/High intake groups included all participants who reported eating legumes, which was about 24% of the sample (n = 3614), and these two groups were split precisely in half, so the Low group included 12% of the sample and the Moderate/High category also included 12% of the sample.

Fiber Intake. Dietary fiber was employed as a covariate to determine the extent that the relationship between legume intake and weight-related outcomes was influenced by differences in dietary fiber. Grams of soluble fiber and insoluble fiber were included in the total. Total dietary fiber was expressed as grams consumed per 1000 kcal.

Energy Intake. Total energy (kcal) consumption was based on all foods and beverages consumed during the previous 24 h (midnight to midnight). Energy intake was employed as a reference variable for legume and fiber consumption, as both were reported in grams per 1000 kcal. Participants who significantly underreported their energy consumption were not included in the sample. Specifically, under-reporting was defined as intakes that were 3 or more standard deviations below the mean (<492 kcal).

10-Year Percent Weight Change. A digital scale was used to weigh subjects in kilograms (kg). The scale was checked each day using precise weights. A standard paper gown was worn during the assessment. It included disposable pants, a shirt, and slippers. Under the gown, underwear was worn. To assess 10-year weight change, subjects reported their body weight from 10 years previous. That weight was subtracted from their present weight. Percent weight change was then calculated by dividing the difference between the two weights by the initial weight. For example, if a subject’s current weight was 90 kg and initial weight was 80 kg, then the percent weight change was (90 − 80)/80 = 12.5%.

According to a number of high-quality studies, self-reported body weight is closely related to measured weight. In a study by Stunkard et al. [27] using more than 1300 men and women, the two measures were almost perfectly correlated (r = 0.99, *p* < 0.01). Stunkard concluded, “Self-reported weights were remarkably accurate across all these variables in the American sample, even among obese people, and may obviate the need for measured weights in epidemiological investigations” (p. 1593). Similarly, investigations by other researchers studying the relationship between self-reported and measured body weights have resulted in strong correlations (r = 0.98) [28].

Studies comparing current body weights to recalled weights of the past have also produced very similar values. For example, when subjects were asked to remember their draft registration weights at age 25 when they were 45–55 years old, on average, the difference was only 2.2% [29]. Likewise, in a large sample, women were required to remember their body weight at age 18 when they were 25–42 years old. The two weights were highly correlated (r = 0.87, *p* < 0.01) and only differed by 1.4 kg on average [30].

In more recent investigations, Kyulo et al. examined the validity of recalled body weight over a 26-year period in more than 2700 older adults (mean age: 70 years). The average difference was 0.67 kg, and the two weights were highly correlated (r = 0.88, *p* < 0.0001) [31]. The authors concluded that the “validity of 26-year recall of body weight during adulthood was very high in an older sample” [31]. Finally, Tamakoshi et al. compared recalled body weights at 25 years of age with measured weights at age 25 in 2453 men aged 34–61 [32]. The recalled weights were strongly correlated with the measured weights (r = 0.85), and the difference was 1.3 kg.

Willett showed that measured BMIs and recalled values roughly 55 years earlier were correlated 0.92 (*p* < 0.01) in women and 0.91 (*p* < 0.01) in men. He concluded that “recalled weight from many years earlier appears to be highly valid” (p. 216), and differences between recalled and measured values “have minimal effect on epidemiologic measures of association” (p. 216) [33].

Body Mass Index (BMI). As height increases, body mass tends to increase. BMI is the standard index of body mass adjusted for differences in height. BMI was determined using the equation: weight in kilograms divided by height in meters squared, kg/m^2^. BMI allows differences in body mass or weight to be compared independently of stature or height. In the present study, height was measured using a wall-mounted stadiometer.

Waist to Height Ratio (WHtR). Similar to body mass, as height increases, waist circumference tends to increase. Therefore, the present study used waist circumference as a measure of abdominal adiposity corrected for differences in height. This is known as the waist-to-height ratio. It is calculated by dividing waist circumference (cm) by height (cm).

Waist circumference is an excellent predictor of differences in abdominal adipose tissue indexed using magnetic resonance imaging, accounting for 91% of the variance [34]. Numerous investigations indicate that the waist-to-height ratio is a better predictor of cardiometabolic health than BMI and typical measures of waist circumference (WC) [35,36,37]. According to a review and meta-analysis by Ashwell et al., which included over 300,000 individuals from diverse populations, the WHtR is better than BMI and WC at predicting diabetes, dyslipidemia, CVD, hypertension, and all these combined [36].

Waist circumference was assessed by trained technicians. The specialists were graded regularly to ensure the validity and reliability of their measurements. A specially designed room in the mobile examination center was utilized for the measurements. A trained recorder helped with the waist measurements. The superior border of the ilium was used as the reference point for the horizontal placement of the measuring tape. A wall mirror, along with the assistant, was employed to confirm the horizontal placement of the tape. The goal was to place the tape snugly around the waist without compressing the tissue. The waist assessment was taken after a normal exhalation [38].

Smoking. Cigarette smoking was evaluated by asking participants to report the number of cigarettes smoked per day during the past month. Subjects who responded that they did not smoke any cigarettes in the past month were assigned zeros, and smokers were given a maximum of 95 cigarettes per day [39]. Cigarette smoking values were controlled statistically in this investigation.

Physical Activity. Total physical activity was also employed as a covariate. An interview was utilized to assess physical activity. Moderate and vigorous activities were evaluated separately. Activities that caused modest increases in heart rate and breathing speed were defined as moderate. Casual biking and walking were examples of moderate physical activity. On the other hand, significant increases in heart and/or breathing rate were recorded as vigorous physical activity. Jogging, running, or walking up a hill were used as examples of vigorous physical activity.

Specific questions were utilized to ascertain the time spent in moderate and vigorous physical activity, each asked separately. The focus was on “days per week” and “time per day.” Days and minutes were combined using multiplication, resulting in minutes of moderate and minutes of vigorous activity per week. Participants reporting more than 8 h per day of moderate or more than 5 h per day of vigorous activity were given these values, respectively. The moderate and vigorous minutes were added together, producing the total time (minutes) spent in moderate and vigorous physical activity (MVPA) per week.

The NHANES PA questionnaire was based on the World Health Organization (WHO) “Global Physical Activity Questionnaire (GPAQ) [40]”. The U.S. version was adapted for those using the English language. The GPAQ is currently used by over 50 countries to monitor physical activity within their borders. Research shows that the questionnaire results that focus on MVPA are significantly correlated with pedometer counts, Actigraph accelerometer counts, percent body fat, waist circumference, and VO2 max results. Indexed using the intraclass correlation (ICC), the test-retest reliability of the questionnaire across 10-days was 0.96 for moderate recreational physical activity and 0.90 for vigorous recreational physical activity [41].

Alcohol Use. A total of 3 categories were used to differentiate among alcohol users: Abstainers, Moderate drinkers, and Heavy drinkers. To be classified as an Abstainer, participants had to respond that they consumed no alcohol in the past year. For men, Moderate drinkers reported drinking more than 0 and less than 3 alcoholic beverages per day during the past 12 months. For women, Moderate drinkers reported drinking more than 0 but less than 2 drinks per day over the past year. For men, Heavy drinkers reported drinking 3 or more alcoholic drinks per day over the past year, whereas, for women, Heavy drinkers reported drinking 2 or more alcoholic beverages per day over the past year.

Economic Status. Another covariate was economic status. It was assessed indirectly using a question about housing, which placed subjects into one of 3 categories. Specifically, participants were asked if they were renting or buying their dwelling or other.

Race/Ethnicity. To control for differences in race/ethnicity, six categories established by NHANES were utilized: Mexican American, Non-Hispanic Black, Non-Hispanic White, Non-Hispanic Asian, Other Hispanic, or Other Race/Multi-Racial.

### 2.3. Data Analysis

Findings can be generalized to the U.S. adult population because NHANES employed a multi-level, random sampling strategy to secure participants. To achieve this, each statistical model incorporated clusters, strata, and individual sample weights.

Given the very large sample associated with the current investigation, statistical power would be projected to be exceptionally high. Nevertheless, because of the unique multi-stage random sampling approach used by NHANES, the degrees of freedom (df) were established by subtracting the number of strata (59) from the number of clusters (121), resulting in 62 df instead of more than 10,000 df in the denominator.

There were 3 outcome measures (10-year percent weight change, BMI, and WHtR) and 1 exposure variable (grams of legume intake per 1000 kcal). Adjustments were made for differences in 5 demographic covariates (age, sex, race/ethnicity, year of assessment, and economic status) and 5 lifestyle covariates (physical activity, cigarette smoking, alcohol use, energy intake, and fiber intake per 1000 kcal) to minimize their effect on the associations between legume intake and the outcome variables. Special attention was focused on the effect of controlling for differences in fiber intake on the relationships of interest.

The associations between legume intake expressed as a categorical variable with 3 levels (None, Low, and Moderate/High) and the 3 outcome measures, each a continuous variable, were evaluated using analysis of variance (ANOVA) and multiple regression. The SAS SurveyReg procedure was used to evaluate mean differences across the legume categories, taking into account the NHANES sampling weights. The covariates were controlled statistically using partial correlation, and adjusted means were compared across the 3 legume intake categories. The Variance Inflation Factor (VIF) was evaluated because of the possible issue of multicollinearity with fiber consumption and legume intake. VIF was 1.9 or lower, so multicollinearity was not a threat. SAS version 9.4 (SAS Institute, Inc., Cary, NC, USA) was used to analyze the data. Two-sided statistical tests were employed, and *p*-values were set at <0.05 to operationalize statistical significance.

## 3. Results

A total of 10,137 participants were included in the 10-year weight change calculations, and 15,185 were integrated into the cross-sectional analyses. The median age of the U.S. sample was approximately 45 years, and about 50% of the representative sample reported that they engaged in moderate or vigorous physical activity for about 1 h and 13 min or more of per week. Percentiles for each of the principal continuous variables are shown below in Table 1.

Table 1 indicates that U.S. adults gain significant amounts of weight over time. About 50% of U.S. adults gained more than 5% in body weight during the previous decade, whereas one in four adults gained almost 15% during the previous 10 years, and 10% gained more than 25% in weight. Additionally, fiber intake was low. As displayed in Table 1, the median U.S. adult intake was 7.8 g of fiber per 1000 kcal, and the 90th percentile was only 13.5 g per 1000 kcal per day.

Table 2 illustrates that most U.S. adults eat few if any, legumes. Over the two days of dietary evaluation conducted by NHANES, intake was 0 g per 1000 kcal for about 76% of the U.S. population. Table 2 also indicates that almost half of U.S. adults reported that they abstain from drinking alcohol, and 48% reported that they own or are buying their dwelling place. Furthermore, by design, about 25% of adults participating in the NHANES survey were selected from each two-year data collection cycle between 2011 and 2018, and the present sample included a wide range of racial/ethnic groups, with nearly equal representation from women and men.

As shown in Table 3, legume intake was a strong predictor of 10-year weight change. There was a consistent inverse dose-response relationship. As legume intake (grams per 1000 kcal) increased, the 10-year weight gain decreased. Adults with moderate to high intakes gained substantially less weight over the past decade than those reporting no legume intake. They also gained significantly less than adults reporting low consumption of legumes. Specifically, adults reporting no legume intake gained 10.5% of their initial body weight over the previous 10 years, whereas moderate/high consumers gained 8.5%. Percent weight gain was 23.5% greater in the non-consumers compared to the moderate/high consumers.

Adjusting for differences in the demographic covariates, age, sex, race, year of assessment, and economic status, as well as the lifestyle covariates, physical activity, smoking, alcohol use, and energy intake, had little effect on the findings. However, when dietary fiber intake (grams per 1000 kcal) was controlled with the other covariates, the association between legume consumption and 10-year percent weight change was nullified.

The cross-sectional analyses revealed similar findings to the 10-year results. With BMI as the outcome variable, there was an inverse dose-response relationship. As legume intake increased, BMI levels decreased. Specifically, women and men reporting no legume consumption had significantly higher BMI levels than their counterparts. Controlling for the lifestyle covariates along with the demographic factors had little influence on the mean differences. However, when adjustments were made for fiber intake and other covariates, BMI differences across the groups were no longer statistically significant.

With the waist-to-height ratio employed as the outcome variable, again, the association was inverse and dose-response. U.S. men and women who consumed legumes had less abdominal adiposity compared to those reporting no legume intake. Moreover, as legume consumption increased, the waist-to-height ratio tended to decrease. Controlling for the covariates had almost no influence on the mean differences. However, like the other associations, adjusting for differences in dietary fiber intake eliminated the relationship between legume consumption and abdominal adiposity.

Not shown in the table, legume intake was significantly related to fiber consumption. Specifically, with all the covariates controlled, mean fiber intake differed across the three legume categories (F = 174.1, *p* < 0.0001). Subjects reporting no legume intake averaged (±SE) 8.1 ± 0.1 g per 1000 kcal, whereas those with Low intakes averaged 9.6 ± 0.2 g per 1000 kcal, and U.S. adults with Moderate/High legume intakes averaged 11.5 ± 0.2 g of fiber per 1000 kcal. The dietary fiber difference between the 1st and 3rd categories of legume intake (11.5 vs. 8.1) was approximately 42%.

## 4. Discussion

The fundamental objective of the present study was to measure the relationship between legume intake and 10-year percent weight change in a large sample of randomly selected U.S. women and men. Additionally, identifying the cross-sectional associations between legume consumption and BMI and also abdominal adiposity were important aims. Finally, another key purpose was to determine the extent to which various covariates, particularly fiber intake, influenced these associations.

This study had four findings worthy of highlighting. (1) Ten-year percent weight change differed significantly and in a dose-response pattern across the three legume intake categories. (2) Cross-sectionally, mean levels of BMI and abdominal adiposity also differed significantly and in a dose-response manner across the categories of legume consumption. (3) Adjusting for nine demographic and lifestyle covariates had little effect on the legume relationships. (4) Controlling for differences in dietary fiber intake with the other covariates negated the associations between legume intake and the weight-related outcomes for the 10-year results and the cross-sectional findings.

The consistency of the key findings was notable. Whether the focus was on 10-year changes in weight or cross-sectional findings associated with BMI and abdominal adiposity, the results were similar: legume intake was a good predictor of the outcomes. Additionally, in each case, controlling for differences in fiber consumption nullified the relationships.

The average 10-year weight gain in this U.S. representative sample was high, but it was significantly higher in adults who did not consume legumes compared to their counterparts. For example, 10-year percent weight gain for adults reporting no legume intake was 10.5% (see Table 3) or about 9.45 kg (20.8 lbs) for a 90 kg (198 lb) person. On the other hand, participants reporting Moderate/High legume consumption gained 8.5% or 7.65 kg (16.8 lb) for a 90 kg (198 lb) person. Over 10 years, the difference was meaningful. Across several decades, the weight gain difference could be substantial.

Literature reviews indicate that the vast majority of randomized controlled trials have not found that regularly eating legumes results in weight loss [2,14]. Part of the problem could be that many legume intervention studies have employed small sample sizes. For example, researchers have conducted experiments focusing on legume intake and weight loss using 18 men [42], 20 men [43], 19 adults [44], and 21 men [45]. Unless within-group variation is minimal, more participants are usually required to generate enough statistical power to detect the mean differences between groups when small samples are employed.

Another issue that might partly explain the lack of significant treatment effects could be the duration of legume interventions. Weight loss is not easy for most adults. It takes time and practice. When legume-based interventions last only 3 weeks [43,46], or 4 weeks [47,48], or 5 weeks [49], weight loss differences between treatment and control groups are difficult to generate, especially when energy intake is designed to be iso-caloric, which many legume investigations have used [14].

Although randomized controlled trials are considered the gold standard by many, they have a few significant drawbacks. Treatments are often limited in duration and magnitude, so compliance does not suffer. Participants are usually unwilling to follow a special diet for long periods. Moreover, some subjects may not appreciate or tolerate eating large quantities of legumes. Many legume-focused randomized controlled trials have required participants to eat 250–275 g or more of legumes daily [43,45,50,51], so dietary compliance could be an issue. On the other hand, the present investigation did not require subjects to eat a particular diet. Instead, the usual diets of participants were assessed. Therefore, conformity to dietary treatment requests was not an issue, and reported intakes in the present investigation might better reflect long-term dietary patterns than those achieved with intervention studies. Other observational investigations have also found this to be the case [16].

Why would legume intake be a good predictor of 10-year weight change? What factors might account for the differences in BMI and waist-to-height ratio? There are multiple possible mechanisms. For example, legumes contain a significant amount of plant protein. As a result, legumes have a dual classification. They are officially a vegetable and also a protein food according to the U.S. Dietary Guidelines for Americans, 2020–2025 [52]. No other food group has this characteristic. Several investigations have determined that plant protein consumption reduces the risk of obesity [53,54].

Moreover, legumes tend to be low in fat. Dietary fat is energy dense, providing more than twice the energy per gram than carbohydrate and protein. In other words, gram for gram, legumes tend to be low in calories. Numerous investigations indicate that regular intake of low-fat foods decreases the risk of weight gain [55,56,57,58].

Additionally, legumes have a favorable impact on the gut microbiome [59,60]. A healthy digestive environment has been shown to diminish the development of several diseases, including obesity [61]. Regular intake of legumes tends to improve the gut microbiome, which could assist with maintaining a healthy weight.

Overall, legumes are a low glycemic index food [62]. That means that blood glucose concentrations are not raised by eating legumes compared to most carbohydrates. Legumes are among the foods that are encouraged to help manage glucose concentrations [13]. A number of investigations have shown that dietary patterns that focus on low-glycemic foods, such as legumes, reduce the risk of obesity and abdominal adiposity [63,64].

Further, some foods satiate better than others. Consumption of legumes tends to appease hunger and encourage cessation of eating. Multiple studies have found that eating legumes leads to consuming consumes less energy than other foods [65]. A meta-analysis of nine experiments showed that legume intake improved short-term satiety by over 30% when matched with other dietary choices [66].

Last, and perhaps of most importance, legumes are an excellent source of dietary fiber [67]. The present investigation targeted dietary fiber intake to determine its effect on the predictive utility of legumes. According to the USDA, legumes provide roughly 12–16 g of fiber per cup [12]. In contrast, a cup of cooked white rice contains less than 1 g of dietary fiber [12]. The high fiber content of legumes could be the driving factor accounting for the strong relationships found in this study. Dietary fiber is essentially calorie-free, but it helps to satiate. Moreover, soluble fiber can bind with fats and sugars and reduce their absorption and use in the body.

Adjusting for differences in dietary fiber intake not only caused the significant weight-related differences between the legume intake groups to be nullified, but it also had other meaningful statistical effects. First is the F-ratio, which reflects the magnitude of the between-group differences (variance) over the within-group variance (error). For the models focusing on 10-yr percent weight change, Model 2 (which included all the covariates except fiber intake) showed an F-ratio of 6.5. When fiber intake was added to Model 2 (thus becoming Model 3), the F-ratio decreased to 1.9, a decrease of more than 3-fold. For the Body Mass Index, the decrease in the F-ratio was 10-fold, another large and meaningful decrease. For the waist-to-height ratio, the decrease was almost 8-fold. Overall, controlling for differences in fiber intake led to substantial changes in the magnitude of the ratio of the between-group variance over the within-group variance.

For the 10-yr percent weight change variable, after adjusting for all the covariates (except fiber intake), the mean difference between the two extreme legume intake groups (None vs. Moderate/High) was 2 percentage points of body weight. That is a meaningful difference in weight gain, especially when considering the size of the samples and that the weight gain difference was associated with just one variable: legume consumption.

Also meaningful is the fact that the weight gain difference between the two legume intake groups (None vs. Moderate/High) was decreased by 50% when fiber intake was added to the variables controlled in the model. Similarly, when focusing on the waist-to-height ratio variable, the weight gain difference between the two legume intake groups was decreased by over 90% when fiber intake was added to the other covariates. It appears that many of the differences in weight change, BMI, and abdominal adiposity between the legume intake groups were a function of differences in fiber intake.

Studies consistently indicate that a high-fiber diet decreases the risk of overweight and obesity and promotes a leaner body [68,69,70]. In the current investigation, subjects in the Moderate/High legume intake category consumed 11.2 g of fiber per 1000 kcal per day. Those in the middle group averaged 9.6 g, and adults reporting no legume intake ate 8.1 g of fiber per 1000 kcal per day (F = 174.1, *p* < 0.0001). Clearly, legume intake goes hand-in-hand with fiber intake.

Findings indicated that fiber consumption in the U.S. adult population is far below the levels recommended in the Dietary Guidelines for Americans (2020–2025) [52]. Specifically, results (Table 1) showed that the median U.S. adult intake is about 7.8 g of fiber per 1000 kcal. The latest Guidelines recommend that adults eat 14 g or more per 1000 kcal. Therefore, most U.S. adults are consuming at least 79% less dietary fiber than they should be. This could be one reason weight gain and obesity are serious issues within the U.S.

There were several limitations connected with this investigation. It was an observational study. There was no treatment or intervention. Hence, causation cannot be inferred. Regular consumption of legumes could result in less weight gain over time and lower levels of BMI and abdominal fat. However, other factors besides legume consumption could account for the differences discovered in this investigation. Similarly, eating legumes regularly might be an indicator of other behaviors or conditions that influence weight gain. Ten covariates were controlled in this study. There are always others that could account for some of the relationships identified in this study. Additionally, legume consumption was assessed using two 24-h dietary recalls. More recall assessments would have been better and would have reduced the misclassification of subjects within the legume intake categories. Moreover, weight change in the present investigation was partly based on weight recall. Although many investigations have found weight recall to be accurate and valid, the recall includes more errors than the direct measurement of body weight. Overall, reduced measurement error from the two dietary assessments and the weight recall evaluations would have likely increased the magnitude of the weight-related differences between the legume intake groups because error variance does not predict well.

The current investigation also had many merits. There were more than 15,000 participants. Individuals were chosen randomly, so the findings apply to the non-institutionalized U.S. adult population. Six racial/ethnic groups were sampled, and women and men 18–75 years old were included, so participants represented a broad spectrum of the United States. Legume intake was measured using the average of two, not just one, 24-h dietary recall assessments. The technicians and specialists who worked directly with the participants were well-trained. They were evaluated regularly and recertified systematically to ensure that high-quality measurement methods were practiced.

## 5. Conclusions

The scientific literature indicates that legumes are a special food. They have an array of characteristics that make them nourishing and healthful. Legumes are full of nutrients, but they are not calorically dense. Despite their many qualities, they are not eaten regularly by Americans. The present study found that U.S. adults who include legumes in their diets have significantly less 10-year weight gain than their counterparts and lower BMIs and leaner waists. Overall, it appears that legumes have dietary attributes associated with successful weight control in U.S. adults.

## Figures and Tables

**Table 1:** Percentile distributions of the key continuous variables representing U.S. adults (n = 15,185).

Variable	Percentile				
	10th	25th	50th	75th	90th
Age (years)	22.5	30.6	44.7	57.3	66.0
10-year weight change (%)	−10.6	−2.9	5.5	14.6	25.9
Waist to Height Ratio	0.47	0.52	0.58	0.65	0.73
Body Mass Index (kg/m^2^)	21.8	24.3	28.1	33.0	38.4
Energy intake (kcal)	1244	1574	2009	2553	3154
Fiber intake (g/1000 kcal)	4.4	5.8	7.8	10.4	13.5
Physical Activity (MVPA: min/week)	0	0	73	239	479
Cigarettes (per month)	0	0	0	0	290

Note. Table values include person-level weighted adjustments based on the sampling methods of NHANES, so values represent those of the U.S. adult population.

**Table 2:** Characteristics of the sample based on weighted values for each column for the categorical variables (n = 15,185).

Categorical Variable	N	%	SE
**Sex**			
Women	7608	50.1	0.56
Men	7577	49.9	0.56
**Race/Ethnicity**			
Mexican American	1473	9.7	0.98
Other Hispanic	957	6.3	0.60
Non-Hispanic White	9657	63.6	1.75
Non-Hispanic Black	1716	11.3	0.98
Non-Hispanic Asian	835	5.5	0.48
Other Race/Multiracial	547	3.6	0.28
**Year of Assessment**			
2011–2012	3720	24.5	1.28
2013–2014	3766	24.8	1.34
2015–2016	3796	25.0	1.18
2017–2018	3903	25.7	1.03
**Economic Status (housing)**			
Renting	3584	23.6	0.99
Buying	7289	48.0	1.54
Other	4312	28.4	1.08
**Alcohol Use**			
Abstainer	7046	46.4	1.12
Moderate Drinker	3872	25.5	0.92
Heavy Drinker	4267	28.1	0.65
**Legume Intake**			
None	11,571	76.2	0.66
Low	1807	11.9	0.43
Moderate/High	1807	11.9	0.52

Note: SE refers to the standard error of the percentage. The N and % columns refer to the number of subjects and the percentage of participants after the NHANES sample weights were applied. Weighted values are more meaningful than unweighted values because they can be generalized to the U.S. adult population.

**Table 3:** Mean differences in 10-year percent weight change, BMI, and the waist-to-height ratio, given 62 degrees of freedom, across categories of legume intake, after adjusting for the covariates.

	Legume Intake (grams/1000 kcal)							
	None		Low		Moderate/High			
Outcome Variable	Mean	95% CI	Mean	95% CI	Mean	95% CI	F	*p*
**10-yr Percent Weight Change**	n = 7673		n = 1231		n = 1233			
Model 1	10.5 ^a^	9.6–11.4	9.9 ^a^	8.3–11.5	8.5 ^b^	7.5–9.6	6.7	0.0023
Model 2	10.5 ^a^	9.6–11.3	9.7 ^a^	8.2–11.3	8.5 ^b^	7.4–9.5	6.5	0.0028
Model 3	10.3	9.4–11.1	9.9	8.4–11.5	9.3	8.3–10.3	1.9	0.1626
**Body Mass Index**	n = 11,571		n = 1807		n = 1807			
Model 1	29.7 ^a^	29.4–29.9	29.1 ^b^	28.6–29.7	28.9 ^b^	28.5–29.4	7.2	0.0015
Model 2	29.5 ^a^	29.3–29.8	29.0 ^b^	28.4–29.5	28.8 ^b^	28.4–29.3	8.0	0.0008
Model 3	29.5	29.2–29.7	29.1	28.6–29.7	29.3	28.9–29.8	0.8	0.4399
**Waist to Height Ratio**	n = 11,571		n = 1807		n = 1807			
Model 1	0.601 ^a^	0.596–0.605	0.590 ^b^	0.582–0.598	0.589 ^b^	0.582–0.595	9.7	0.0002
Model 2	0.598 ^a^	0.584–0.612	0.589 ^b^	0.573–0.604	0.587 ^b^	0.572–0.602	9.4	0.0003
Model 3	0.599	0.592–0.606	0.593	0.584–0.603	0.598	0.588–0.607	1.2	0.3221

Means on the same row with the same superscript letter are not significantly different. Means have been adjusted based on the covariates in the model. Model 1 included age, sex, race, year of assessment, and economic status as covariates. Model 2 included the covariates of Model 1 and also minutes of moderate to vigorous physical activity, number of cigarettes smoked per month, total energy intake, and alcohol use. Model 3 included the same covariates as Model 2 and also fiber intake per 1000 kcal. For the 10-yr weight change variable (Model 3) the mean difference between Low and Moderate/High intake was borderline significant (*p* > 0.05, *p* < 0.10).

## Data Availability

All data sets associated with the results of the present study can be found online as part of the National Health and Nutrition Examination Survey (NHANES). There is no charge for the data sets. They can be downloaded at the following website: https://wwwn.cdc.gov/nchs/nhanes/Default.aspx (date accessed: 5 January 2023).
